# Book Reviews

**Published:** 1810-11

**Authors:** 


### CRITICAL ANALYSIS

OF

### RECENT PUBLICATIONS

IN THE

### DIFFERENT BRANCHES OF PHYSIC, SURGERY, AND MEDICAL PHILOSOPHY;

---

*A Dissertation on Retroversion of the Womb, including some Observations on Extra-Uterine Gestation. By* SAMUEL MERRIMAN, M. D. 8vo. Lond. 1810. pp. viii—80. Callow,

THE substance of this dissertation originally appeared in the volumes of this Journal. In its present dilated and improved form, it is intended to establish two points of importance in the theory of Pregnancy, and the practice of Obstetrics.

Retroversion of the uterus, as a fact occurring at one period of uterine gestation, is well known : the extension of this displaced position of the womb to the last term of pregnancy, is not, however, so fully ascertained ; neither has it hitherto been subjected to much investigation. Dr. Merriman adduces several cases to prove the existence of this state of the uterus in the latter months of gestation, and that it also gives rise to another fact in the history of pregnancy, commonly denominated *extra-uterine gestation ;* but which has, from its discordance with established physiological principles, remained doubtful, obscure, or " incomprehensible."

That retroversion of the uterus must have occurred at all periods, cannot be doubted ; but that its real phenomena have only lately been understood, occasions more regret than surprize. Neither Mauriceau, Lamotte, nor Deventer, who attended particularly to a state of the womb, which we denominated its obliquity,* had any precise knowledge of this altered position of the uterus, nor of the effect it produced on the bladder and rectum ; deranging the functions, and obstructing the operations of these organs. " The first person who entertained any " correct idea of this accident, was M. Gregoire, an accoucheur of considerable reputation at Paris, about the middle of the last century, and " a lecturer on the practice of midwifery. M. Gregoire's lectures were " attended by many students, who afterward became celebrated accoucheurs ; and among others our countryman *Smellie :* yet his doctrine " upon this subject seems to have been recollected by only two of his " pupils,—the French accoucheur M. *Levret,* and Mr. *Wall* ; and from " the latter the accoucheurs of England first gained information on this " subject."

---

* *Rien ne lui fit plus d honneur,* says his biographer, *que d'avoir prouve que l'obliquité de la matrice est une des primicres causes des accouchemens difficiles, & d'avoir indiqué la manœuvre que demandent les accauchemens de celte espece.*

" The

"The lectures which Gregoire delivered were never printed, and "therefore it is impossible to know exactly what opinion he had formed "of this accident; but that he was aware of the *fundus uteri* being "thrown down between the vagina and rectum, is evident from the di- "rections which he gave for replacing it.* In the year 1754, Mr. Wall, "who had settled in London, was called to attend a woman labouring "under a suppression of urine, from a retroversion of the uterus, and "being convinced that it was a case of the kind described by Gregoire, "he endeavoured to relieve his patient by the method taught by that "lecturer; but failing in his object, he called in the assistance of the "late Dr. William Hunter. Their united endeavours in various ways "were unavailing, and the woman died! Dr. Hunter, with the most "laudable desire to disseminate knowledge, having obtained leave to "open the body, invited a number of gentlemen of the faculty of me- "dicine to a public lecture, which he read for the purpose of making "the disease generally known. Drawings were taken of the parts, "from the dissection of this woman, and an engraving from these was "inserted in Dr. Hunter's splendid *Anatomy of the Human Gravid* "*Uterus*." This case, however, did not come fully before the public, until in 1771 it was printed in the 4th volume of the Medical Observations and Inquiries (p. 400), as an appendix to a history of retroverted uterus, there related by Mr. Lynn, of Woodbridge in Suffolk. Mr. Lynn's narrative is, we apprehend, the first printed account of this form of retroverted uterus published in England.

From the year 1771 this accident has so often been described, that its symptoms are perfectly understood, and are relieved with great certainty by the simple process of introducing the catheter so frequently as to prevent the bladder being distended with urine.† For many useful ob-

* M. Gregoire instructed his pupils, that with one finger in the vagina and another in the rectum, an attempt should be made to raise the fundus and to replace the uterus. This was advised to be done while the woman was lying on her back, an unfavourable position for the purpose: for, should this manœuvre ever be deemed necessary, the objects of it may be accomplished with more facility while the patient is resting on her elbows and knees.

† In the cure of retroverted uterus, the principal reliance is to be placed upon the introduction of the catheter; and this should be done twice at least in twenty-four hours. Care is likewise to be taken to keep the bowels open, and rest is to be enjoined. By pursuing this plan steadily, the mal-position of the uterus is usually overcome in a few days. It is still customary with some practitioners of eminence to make use of artificial means for replacing the womb, after the bladder has been emptied and the bowels opened; and there can be no great objection to making such an attempt, provided it be done cautsously, and that no (improper) force be made use of. In general, however, nothing of this kind is either necessary or advisable." p. 24.

In many cases of retroverted uterus which have fallen under our care, the catheter thus employed, enjoining a recumbent posture, and attending to the state of the bowels, has been completely successful. In some of these cases the uterus was alarming impacted in the pelvis; yet the termination was always fortunate. The introduction of the catheter, and the emptying the bladder by that means, being the desideratum in the management of this complaint, the following practical observations by *Dr. Denman* cannot fail to be acceptable. "It cannot be disputed (*Introduction, vol. i.* 140.) but that all attempts to restore the *uterus* to its natural position, before the distention of the bladder is removed, must be fruitless, servations on this form of the disease, we are compelled, though unwillingly, to refer to the Dissertation itself; and can only remark, that retroversion has also recurred in the unimpregnated state of the uterus, when it has been enlarged by disease. Of this an instance is related by Dr. Merriman, and Mr. John Pearson (Observations on Cancerous Complaints, 8vo. Lond. 1723), on examination, in a case of diseased uterus, "found it hard, immoveable, and so much enlarged, that it "filled up the superior part of the vagina: it was also retroverted, the "*os uteri* pressing against the bladder, and the *fundus uteri* compressing "the rectum." p. 114. In these cases, however, it is obvious that the danger, as arising out of the position of the uterus, will neither be so great, or approach with such rapid strides, as in the impregnated state of that organ.

The continuance of the impregnated uterus in a retroverted position to the last period of utero-gestation, is a fact possessing both novelty * and interest; especially as Dr. Merriman deduces conclusions from it, explanatory of what has been deemed *extra-uterine gestation*.

Of this state of the uterus Dr. Merriman relates two instances; one of them is that which occurred to Dr. Jackson, as specified in the note below, and the other is a minutely circumstantial history of a case which happened in 1806. It is hardly to be doubted, however, that this variety of retroverted uterus, differs very essentially from that which occurs in the early period of pregnancy. In the mal-position of the uterus, prior to the rising of that organ above the brim of the pelvis, the danger is hourly increasing by the increasing size of the uterus while impacted in that bony cavity; and by which its pressure, both on the bladder and rectum, is carried to a point that completely obstructs the expulsion of their contents. In the other variety, that which continues to the full term of utero-gestation, the womb being necessarily above the brim of the pelvis, and lying among parts that may recede from its pressure, though much inconvenience will arise, no urgent danger presents till the period of parturition. We mistake Dr. Merriman exceedingly, if he does not lead his readers to understand that this second variety, or that which lasts to the end of utero-gestation, is a continuance of the first variety, or the retroverted uterus of

---

fruitless, as the *uterus* will be irresistibly borne down by the pressure of the superincumbent *bladder*. The first step then to be taken for the relief of the patient is, to draw off the urine; yet there is always in these cases great difficulty in the introduction of the common catheter, because the urethra is elongated, altered in its direction, and pressed against the ossa pubis by the tumour formed by the retroverted uterus; and many women when the *uterus* was retroverted, have lost their lives from the want of expertness in introducing the *catheter*. But the attending inconveniences may be avoided or surmounted by the use of a flexible *catheter*, slowly conducted through the *urethra*. I say slowly, because, whatever *catheter* is used, the success of the operation, and the ease and safety of the patient, very much depend on this circumstance. The *catheter*, when introduced, should not be carried farther into the bladder, when the urine begins to flow, unless it ceases before the distention is removed; which in some cases happens in such a manner, as to give us an idea of a bladder divided into two cavities."

* We have much satisfaction in stating, that our friend Dr. H. J. Jackson, of Hanover-street, in a judicious little work, (*Cautions to Women respecting the state of Pregnancy, &c.* 8vo. Lond. 1798,) published the first case of this disease.

early early pregnancy; and that it is one of its terminations.* "It has been," he says, "the almost uniform opinion of writers on the retroversion of "the gravid uterus, that a suppression of urine must necessarily be produced by this unnatural situation of the womb, and that this suppression must be removed by art; otherwise, they say, abortion will take "place, or a still more melancholy event, mortification, or rupture of "the bladder, and, consequently, the death of the patient will ensue; "and, in cases where suppression of urine has come on, this has unquestionably *been the usual progress* and termination of the complaint, "unless proper means of cure have been had recourse to at the commencement of the disease. It is, however, consolatory to know, that, "under some circumstances, *the uterus may remain in a state of retroversion* for a very great length of time, even to the completion of the "period of utero-gestation, without producing a total suppression of "urine, or any other very uncommon or alarming symptoms." (p. 25.) It is probable, however, and it is a fact of some importance both as to the true history of the disease and to the practice, that these are two distinct complaints; one being necessarily confined to the first period of utero-gestation, that is, before the uterus rises above the pelvis; the other, as necessarily occurring in that period when, in its enlarged state, the womb has risen into the cavity of the abdomen: one being accompanied with immediate and urgent danger, the other with injurious consequences remote and uncertain. It does not appear that those who have suffered under that variety of retroverted uterus which continues to the time of parturition, have experienced the symptoms and the hazards of that which appears in early pregnancy. "Mrs. F——— did not "suffer more, during her pregnancy, than most other women, except, "that for the *last two or three months*, she was troubled with difficulty "in parting with her urine, and considerable pain in the act of passing "it; yet her sufferings in this particular were not so great as to induce "her to consult her accoucheur on the subject. She neither at this "time, nor at any earlier period of her pregnancy, experienced a total "suppression of urine, nor does she recollect ever having retained it "long enough to occasion any considerable inconvenience." (p. 28). In Mrs. F———'s case there is a distinct point of time marked for the commencement of her disease. "When about *five months* advanced in "her pregnancy, she was much terrified and affected on hearing of the "sudden death of her aunt; which, as she herself expressed it, *seemed* "*to turn her whole inside upside down.*" Whether the feelings of Mrs. F ——— were, or were not, sufficient to determine that then the altered position of the uterus took place, does not seem of much weight; for it is evident, from the history, that it had not been preceded by

* At page 76, Dr. Merriman puts this past all doubt, for he there positively says, "that the uterus, which has become retroverted in the earlier stages of "pregnancy, *may continue in that state* till the full period of gestation has "elapsed." It is not designed to contend that the two varieties of retroversion may not exist at different periods in the same pregnancy; but if they do exist, that the coincidence is accidental, and that they have no dependence one on the other. In Mrs. F——'s case there is no ground to presume an early retroversion. In the case related by Mr. Kelson there was an early retroversion, but this was completely removed long before the accession of the other disease.

the the unvarying symptoms of the first variety of retroverted uterus. In the case related from *Nicholas Patuna*, the patient, "about six weeks "after conception, was attacked with violent pains in the lower belly, "and was unable to pass her urine, except when lying on her back. "From this time, till she entered the ninth month of her pregnancy, "she suffered much at various times, from pains in the belly, and loins, "and other complaints." These symptoms, though they shew the existence of some morbid change, are certainly no evidence that this change was the first variety of retroverted uterus. Mr. Kelson's case, related in the 11th volume of this Journal, was, in the first instance, unequivocally the retroverted uterus of early pregnancy. It was cured by the usual method (a daily introduction of the catheter); and about the 12th week, the impediment to the flow of urine being removed, *the uterus took pretty much its natural situation.* (p. 52). After this, it appears that the second variety of retroversion took place; but at what period was never determined; nor was it demonstrated that this wrong position of the womb did exist, until after the usual period of gestation had elapsed, when the indications of labour enabled Mr. Kelson to ascertain "that the child was plainly to be felt through the vagina, that "the uterus was not enlarged, but forced upwards and forward, and the "*os tinea quite closed.*"

Under every point in which we have viewed this statement, a conviction has arisen, that the variety of retroverted uterus occurring in the latter months of pregnancy, has no dependance on, or connection with that which appears in the early period; that is, before the fourth month of utero-gestation. With this impression, we regret that the disease which Dr. Merriman has so accurately traced, and from which he has deduced a conclusion greatly interesting to science, should have received ths name of *retroverted uterus.* This we regret because it wants precision—because it confuses it with a disease already well known, and with which, we believe, it has no connection—and because it may, in some instances of the true retroverted uterus, lead to a fatal procrastination.

We intreat Dr. Merriman will consider that these observations are not made with the intent to depreciate his ingenious Dissertation, but in the spirit of scientific investigation, and with the ardent desire to elicit truth.

To whatever degree we may dissent from the opinion, that the deranged position of the womb sometimes met with in the latter months of pregnancy, is a continuation or termination of a case, so fully known under the term RETROVERSION:—or how much soever we may object to the application of the same name to two distinct diseases, we readily admit that the subject has been greatly elucidated by our author; and we believe the following conclusions to be formed on the solid ground of fact and fair reasoning.

Whenever that peculiar state of the uterus which Dr. Merriman denominates retroversion, and which he describes as a continuance of the retroversion of early pregnancy does take place, it may be expected to terminate in one of the following ways.

1st. "The good form of the pelvis, the health and strength of the "mother, and the efficacy and continuance of the pains, may all com- "bine to replace the uterus, and produce a favourable issue.

2dly.

2dly. " The want of some, or all, of these fortunate circumstances, " or injudicious management under the labour, may occasion the woman " to fall a victim to this untoward position of the womb, in the course of " a few days, either by a rupture of the uterus producing speedy death, " or by an active inflammation or mortification of the parts.

3dly. " The uterus being unable to extricate itself out of the aukward " position into which it is thrown, may passively submit to the burthen, " till, by the slow process of ulceration, the fœtus may be excluded " through the rectum or vagina, and the mother remain alive."

In the preceding part of the Dissertation, the observations and facts have been principally employed in elucidating that favourable termination of the disease, which occurs when, from the good form of the pelvis and other circumstances in the patient, the womb is restored to its natural position, and the fœtus is extruded by the proper uterine action. The remaining part enters on a disquisition extremely curious and interesting.

The frequent recurrence in the records of medicine of histories of *extra-uterine gestation* would, it might be presumed, have rendered the fact, great as the violation of a law of nature is, sufficiently distinct and tangible. The investigations to which Dr. Merriman has subjected these histories, has demonstrated, however, that some of them must come under a very different class ; while over the remainder is spread a degree of doubt that renders their character equivocal.

" Mr. Kelson, Mr. Colman, and Mr. Mainwaring, have all denominated their communications,* cases of *extra-uterine fœtus*. If by " this is merely meant that the fœtus, after being brought to maturity, " or nearly so, in the uterus, was excluded from thence into the cavity " of the abdomen, either by a rupture of the uterus, or by an ulcerated " opening through its parieties ; and afterwards, by the same proeess, " discharged through the rectum or vagina, I completely accord in the " same opinion ; but if, as I understand, it is supposed that these fœtuses were actually nourished, and brought to perfection in the cavity " of the abdomen, unconnected with, and perfectly distinct from, that " system of vessels which have been expressly set apart by nature for " this purpose, I must confess myself not at all convinced by these cases " of the existence of a fact, so completely at variance with all we know " of the laws of nature, and of the animal œconomy. I mean not to " deny," he adds, " that fœtuses have been found in the cavity of the " abdomen, entirely disengaged from the uterus and its appendages ; " but I think that in such instances a *rupture* of the womb, of the tube, " or ovarium, or an ulceration through their parieties, had permitted the " escape of a full grown fœtus, and not that the conception had advanced to maturity in a part apparently so ill adapted to such a purpose. Every one of the instances of *extra-uterine gestation* which I " have had an opportunity of perusing, has been deficient in some of " the particulars, or symptoms, which are necessary for confirming the " fact, of a fœtus being nourished in a cavity, and by vessels intended

* Cases given in Dr. Merriman's Dissertation from the Medical and Physical Journal, (vols. 2 and 11,) and from the 2d volume of the Transactions of a Society for the Improvement of Medical and Chirurgical Knowledge.

" for

" for other purposes, and distinct from the uterine system; and at the " same time the case was capable of being explained in a more rational " way."

Dr. Merriman examines, with much acumen, several cases which have been considered by their authors as extra-uterine; and shews, with great force of probability, that they have arisen from a rupture or ulceration of the uterus, consequent to that altered position which he has denominated *retroversion*. We are able to add to the catalogue a well-marked history, where the fœtus escaped from the tube, passed by the medium of ulceration into the rectum, and was expelled, *per anum*; and where the uterus had also taken the position which is considered to give rise to such cases. It occurred to Mr. Giffard the surgeon, and was published in 1730, in the Phil. Trans. vol. 36. p. 435.

" I was sent for about the middle of August," says Mr. Giffard, " to a woman who then judged herself to be between three and four " months gone with child: she had all the symptoms preceding a mis- " carriage, and upon touching I found the *os tineæ* somewhat dilated and " spread, and from whence I concluded miscarriage would ensue; but " I was some time after informed, that although she before believed that " she had miscarried, that she now thought herself quick; as feeling " somewhat to move within her, agreeable to what she had perceived " after former quickenings. Thus it passed on for about six or seven " weeks; in which time she grew much bigger, and the motion more " perceptible; so that there remained no doubt of her being with child. " On the third of October she was seized with violent pains in her " belly and back; which daily increasing, I saw her on the sixth, and " found her labouring under very great pains, and other complaints like " those preceding miscarriage or delivery. To be better satisfied, I " passed up two fingers into the vagina, to examine by the touch, whe- " ther the *os tineæ* began to open. *I there felt a large and unusual full- " ness and tension, which I then judged to be the body of the uterus sunk " low in the vagina, and much distending it, and extending backwards, " and pressing against the rectum, so that the excrements could not pass, " neither could she, from its pressure upon the neck of the bladder, freely " make water. I could not find the os tineæ, although I very carefully " examined; wherefore I then judged that the fundus uteri must have re- " ceded from its natural position, and be bent backward toward the rec- " tum: in which opinion I was the more strengthened, from the fulness I " had observed stretching backward; and therefore concluded that the* os " tineæ *must be very forward: wherefore I endeavoured to pass my fin- " gers between the os pubis and the fulness which pressed against the upper " edge of the said bone. This with some difficulty I effected, and at length, " about two or three inches above the said bone, I felt the* os tineæ *with " the ends of my fingers.* The cause of this situation will more clearly " appear in the pursuit of this account. I ordered her anodine and " quieting medicines to relieve her pain, which she was obliged to repeat " every twelve hours; and sometimes clysters. Thus matters continued " to the 20th of the said month, only that for some days before, a wa- " ter tinged with blood came away, as she imagined, through the *anus*.

" On the 20th her husband came to me, with an account that the " midwife had brought away a fœtus, but could not compleat her busi-

" ness.

"ness. The midwife informed me that a fœtus was protruded through "the anus; and to confirm it, desired me to examine, which I did, and "found the *funis umbilicalis* hanging out about three inches beyond the "anus, and passing up through the same. I passed my fingers by the "string into the anus; where I found, about three inches up, an open-"ing, as I then judged, into the uterus, wide enough to admit the ends "of three or four fingers, and the *funis* passing into it; from whence "I was assured that the fœtus came out that way. The septum between "the anus and vagina was entirely whole. From these appearances I "then concluded that a mortification must have begun in the uterus; "and, from its contiguity, been communicated to the rectum; so that "nature, endeavouring to expel what was contained, and forcing it "against this part already mortified, produced this opening, and the "protrusion of the fœtus through it into the *rectum*.

"There was a large discharge of grumous blood and other substances "through the anus, which continued until the 26th, when the woman "died. The fœtus was perfect in all its parts, but wasted from its be-"ing some time dead."

Mr. *Nourse*, then one of the Surgeons of Bartholomew Hospital, examined the body, and has given an explicit dissection, with drawings of the morbid parts.

The *vagina*, *uterus*, *ligamenta rotunda*, *left ovarium*, *fallopian tube*, and *ligamentum latum* on that side, together with the hypogastric and spermatic vessels of the same side, were in a natural state. The *fallopian tube* on the right side, being traced from the *fundus uteri* almost to the *morsus diaboli*, was found confusedly uniting with, and opening into the *sacculus*, hereafter to be described. The *ovarium* on this side, with the *ligamentum latum*, was dilated into a large *sacculus* * of an irregular form, extending behind the *uterus*, to the posterior parieties of which it adhered, and passing on toward the left, was connected to that part of the *colon* that terminates in the *rectum*, and to the *rectum itself*.

In all its circumstances this case has considerable precision; the exact nature of it is verified by dissection. and the annexed plate, in the volume referred to, renders the whole history very clear.

We cannot close our view of this valuable "Dissertation" better, perhaps, than by quoting from it the practical remarks which the author makes on the management of those heretofore mysterious and mistaken cases.

"Dr. Hall, in his elaborate defence of the Cæsarean operation, con-"sidering all those cases as extra-uterine, recommends to cut through "the back part of the vagina, and to bring the child through the os "externum; and the same operation has been recommended by Dr. "Mackenzie and Dr. Kelly. There is a case quoted by Dr. Hall, "which happened to M. Lauverjat, in which the child was extracted "by an incision into the uterus by the vagina. There is a great want "of accuracy in describing the situation of the womb; but I can only "conceive of it, that it was in a retroverted state; and that the incision

* In this *sacculus* was contained the fœtus, and on the dissection were found in it the placenta and several lacerated membranes; and from it there was a large opening into the *rectum*.

"was made through the posterior part of the vagina into the uterus. "Unless this were the position, I see no reason that there could be for "operating at all. Une femme enceinte pour la premiere fois, et par- "venue au moment d'accoucher, éprouvoit des douleurs si vives, que "Lauverjat voulut s'assurer de l'état des choses. Il fut surpris de "trouver la vulve occupée par un corps qui la remplissoit, et la depas- "soit, et qui cedoit a l'impulsion des doights, excepté dans le tems des "douleurs. En parcourant cette tumeur, il ne trouva à la circonfer- "ence qu'un cul-de-sac de demipouce de profondeur, sans overture, "qui peut permettre la sortie de l'enfant. Des conferes mandés pour "ce cas extraordinaire, voulourent voir aussi comment les choses se pas- "aoit. Ils trouverent sur la tumeur, une dechireure qui interessoit "q'une partie de l'épaisseur de ses parois. Cette dechireure leur parut "le lieu, ou il falloit inciser. L'operation faite, le doigt entra dans la "poche dans laquelle l'enfant etoit contenu. Il sortit beaucoup d'eau "bourbeuse. L'enfant se presenta en franchit l'ouverture, qui venoit "d'être pratiquée, et a laquelle il se fit une petite dilaceration du coté "droit. Lauverjat ayant porté la main dans la poche, ne trouva aucune "trace de col ni d'orifice. Du reste il ne survint point d'accident, et "les ecoulment si firent a travers l'ouverture, qui se ferma par degrés. "Deux mois aprés, le col et l'orifice de la matrice etoient dans leur "état naturel. (*Sabatier de la Medicine Operatoire*, tom. i. p. 816.) "The event of this case would, doubtless, under some circumstances, "justify an operation; but considering the possibility, not to say pro- "bability, of all such cases being retroversions of the uterus, and hav- "ing the knowledge of two which terminated so favourably from the "sole efforts of nature, as Mrs. Walker and Mrs. F——s, it is im- "possible that I can recommend an operation, especially at the com- "mencement of the pains, and while the strength, habit of body, and "general health of the patient are uninjured. So long as these circum- "stances remain propitious, there is great reason to expect that the "pains alone may be able to rectify what is amiss, and to effect the de- "livery *per vias naturales*. The observation of Cicero, *Naturæ soler- "tiam, nulla ars, nulla manus, nemo opifex consequi potest imitando*, "should, as far as possible, be our guide in the practice of midwifery; "in which we find that women are seldom injured by a long continu- "ance of labour pains, provided their spirits are not hurried, nor their "blood inflamed, by injudicious attempts to give assistance, and by the "improper use of cordials and stimulants.

"If, however, nature gives up the point, if it is found that the pains "are unequal to the task of restoring the uterus to its place, and ac- "complishing the delivery of the woman, it remains to be considered, "whether it is not more to the advantage of the patient, that such an "operation should be performed, and that she should by this means be "released from her burthen, than that she should continue in the de- "plorable condition, which attends the slow process of discharging "a fœtus through an ulcerated opening in the vagina or rectum; in "which process there must always be great pain and suffering for many "months; and in which, though several women have passed happily "through it, yet others have perished miserably, exhausted with pain "and debility.

"A justi-

"A justification of such an operation may doubtless be found, and "perhaps it may, in some rare instances, be adviseable; but we know, "from woeful experience, that incisions into the uterus have been so "very generally fatal, (in this country in particular) that the experi- "ment ought never to be tried, while any reasonable expectation can be "indulged of saving the patient by any other means."

---

*Observations on some of the Principal Diseases of the Rectum and Anus, particularly Stricture of the Rectum, the Hæmorrhoidal Excrescence, and the Fistula in Ano. By* THOMAS COPELAND, *Fellow of the College of Surgeons, and Assistant Surgeon to the Westminster General Dispensary,* 8vo. *London,* 1810. *pp.* 100. *Callow.*

MR. COPELAND opens this very interesting subject on the Art and Practice of Surgery, with a sensible and modest Preface, introducing the observations in an order perspicuous and methodical, without calling away the attention by any inducement from the valuable information given in the whole of this work. The contents are useful, true and important. They will furnish the practitioner with such arguments for treating the diseases, of which this pamphlet is the object, that the impression derived from it, must give a strong confidence in practice to him who is fortunate enough to study, digest, and embrace it. Whatever is advanced by Mr. Copeland, the reader will find supported by other authors; and whatever is opposed, will be seen to be a difference in opinion, by which the reader has the choice of forming his own judgment. The selections are arranged in the very manner of the most respectable of the ancient writers, either to corroborate the author's own sentiment, or to shew his variation from their text. We hail the time, and congratulate surgeons upon the event, that in this pamphlet we see not only antient, but living authors, quoted with all becoming respect; we see also in this work the scholar of industry, to whom the languages of the different authors he has referred to are open to his research.

Mr. Copeland has divided this important work into sections. The first of which is, "*Observations on the Stricture of the Rectum.*"

The rectum is here considered by Mr. Copeland, like any other part, subject to the consequences of inflammation, and the case aggravated from the action which that intestine is every day compelled to perform in the expulsion of the fæces. He supposes that obstinate cases of Ileus, and other chronic bowel complaints which resist treatment, have their origin in organic obstruction of the canal of the large intestines. He cites, in confirmation of this, authorities of the highest fame. He proceeds to state that strictures are not usually detected early in these complaints, and frequently not discovered till after death: hence, they have been deemed to be fatal, though the fatality was in the want of discovering them. After Mr. Copeland has given us the benefit of the opinions of most ancient authors, and after he has paid that high tribute to Ruysch which we forbear to quote, he proceeds to inform us of that of the moderns. Dr. Sherwin and Dessault are entitled to high notice for what they have imparted on this subject. And when he has thus disposed disposed of the author to whom he has referred, he concludes with this summary.

"But though those who are afflicted with this disease are sometimes quickly carried off with symptoms resembling those of ileus, this is not the most usual form of the complaint, for it commonly assumes a more chronic character. It attacks people of both sexes, and of almost all ages; but is most common about the middle age, and I think, as far as my experience goes, that women are more frequently affected than men.* The first symptom of the disease is an habitual costiveness; but this is so frequent an occurrence, and produced in so many ways, that it is not likely that the cause should be sought for in an organic affection of the rectum; mild purgatives are resorted to, and the symptom being relieved, the cause is no longer sought after.

"When this has subsisted for some time the patient complains of what is called piles, and what is often really so, as a consequence of obstructed circulation in the parts. The remedies usually given in such cases are applied, sometimes with relief, but more frequently otherwise; and then the good old maxim of the inexpediency of curing piles, perhaps rescues the practitioner from the discredit of failing to relieve his patient. The piles are sometimes removed by ligature, or excision, and this gives a temporary abatement of the most painful symptoms, while the cause of the disease is still unknown.

"In a short time, as the gut continues to decrease in diameter, the efforts to expell the fæces become more violent, and the consequent progress of the disease more rapid. The stools, which have been long evacuated with difficulty, become contracted in size, appearing like earth-worms in their form, or small pellets. In this stage it is sometimes, in the male, mistaken for enlarged prostate gland, but if the finger be introduced into the rectum, the gut will be found either obstructed with small tubercles,† or intersected with membranous filaments;‡ or else the introduction of the finger will be opposed by a hard ring of a cartilaginous feel, composed of the diseased inner membrane of the intestine,§ instead of that regular tumour on the anterior part of the rectum, which is formed by an enlargement of the prostate gland. As the disease advances, the fæces become more fluid, and there is a thin sanious discharge from the anus, accompanied with tenesmus; not however the painful tenesmus of dysentery,‖ but with lest distress and less irritation of the parts than in that disease.

---

* The cases which Mons. Derreçagaix relates in Dessault's journal, are principally women, and the most aged is only forty-six years old. Mr. White, in a paper in the London Medical and Physical Journal, Oct, 1809, remarks, that this disease is most common at the decline of life; but his own experience does not bear him out in the assertion, for of eight cases which he gives, six are under fifty years of age. Mr. White considers the disease as necessarily fatal, but he does not seem to have heard of Mons. Dessault's paper.

† Dessault loc. citat.

‡ Bonetus loc. citat. Case 5 in this book.

§ Morgagni, annulo quasi quodam constringi videretur digitus, loc. citato. Adeo induratum ut anceps hærerem an carnosum an cartilaginosum esset dicendum. Ruysch, t. iv. obs. 95.

‖ Sherwin.

"During

" During this time the constitution suffers so little, that the patient might be supposed, from appearance, to enjoy full health. But the ravages of the disease now begin to be felt in their effect on the general habit. Frequent eructations of air confined in the intestines, added to the other symptoms, torment the patient, and render his life miserable. This symptom is so constant, that if it did not occur also in affections of the kidnies and other complaints, it might be regarded as pathognomonic; but I think it prevails to a greater degree in this than in any other disease.

" At this period abscesses very frequently form in the neighbourhood of the anus, and sometimes break into the vagina in the female, and the fæces are discharged through the fistulous orifice. In the male, an adhesion takes place with the bladder and the abscess* discharges itself with the urine, and sometimes fæces and wind are voided by the urethra. But more frequently the matter makes its way through the nates, as in cases of common fistula, for which disease it is not unfrequently treated.

" The patient often continues a long time in this distressing situation, for none of the vital organs are affected, till, at last, worn out with the pain and the discharge, or perhaps the total obliteration of the rectum, he yields to his fate. This is usually the progress and issue of the disease, when it is not early discovered, and I must confess also, sometimes the termination when it is; that is, when the parts are attacked with cancerous ulceration. But I believe that, when the cause of the complaint is ascertained in its early stage, the resources of the healing art are sufficient very materially to relieve, and often to cure it altogether; subject, however, like strictures in the urethra, to the necessity of now and then passing a bougie for a considerable time after the symptoms are removed.

* The rapid progress of disease, from a very trivial origin, in parts subject to continual motion, is very remarkable, in many instances, besides that one under consideration; for this reason, a simple wound near the organs of deglutition is so difficult to heal; for this reason perhaps it is that pulmonary consumption is so fatal a complaint, for the parts being necessarily in constant motion, have not the opportunity afforded them of recovering from an attack of disease."

The second Section is, " *On the Causes and different Kinds of Strictures of the Rectum.*"

In the treatment of this, as in the former section, he refers back into the researches of the Antients; establishes his foundation upon this classic ground, and adroitly concludes in the following manner:

" First, it may be remarked, that we meet with no description of the disease, in any author that I can find, before the time of Wiseman; and

* Petit Œuvres Posthum. tom. ii. p. 93, the contrary also occasionally takes place, and urinary calculi are voided by the rectum. See Paulus Ægineta, and Memoirs of the Medical Society, vol. iii. 496, 542.

† See some very important observations on this subject in Mons. David's Memoir on the Effects of Motion and Rest, translated by Justamond. Also, Mons. Bazille sur les Effets des contre Coups en divers parties du Corps. Prix de l'Academie de Chirurgie, tom. iv. quarto.

the the accuracy of the antient writers of surgery, in their history of diseases, is universally allowed, whatever may be said of some of their modes of cure. It may therefore, I think, be fairly inferred, that either the complaint did not occur so frequently formerly, or that it was overlooked by physicians and surgeons, who suffered scarce any thing which related to diseases altogether to escape them. The case in Wiseman, indeed, was the consequence of the operation for the fistula in ano, as it was then performed; but, I believe, he was the first who has described any species of the disease, and he treated it in a way that was well worthy of the imitation of his successors, if they had sufficiently considered it: but I shall say more of this when I come to the treatment of the disease.

"Soon after Wiseman, as I have related, many authors have described the disease, and they all concur in considering it as inevitably fatal, until Mons. Petit* gives a hint, that when it was a venereal symptom it was curable, by the usual remedies for that disease; but that in other cases he had never seen it relieved.

"Dessault saw it so frequently in combination with other symptoms, decidedly venereal, that he did not hesitate at once to put his patients under a course of mercury, and with a success that fully warrants us in considering it as, very frequently, a symptom of the venereal disease. He did not, however, trust solely to the effects of this remedy for a cure, for he saw that the particular functions and morbid alteration of the part, required a particular local treatment, independent of the medicine which was to cure the constitutional disease. He therefore, during the exhibition of mercury, every day introduced into the rectum tents imbued with some ointment, of a greater or less size, and for longer or shorter time, according to the circumstances of the case, and by this combined local and constitutional treatment generally cured his patient.

"Mons. Dessault does not seem, indeed, to consider the tent of so much importance in itself, but only as the vehicle of his medicaments. Be this as it will, however, he relieved his patients of a disease, which, till then, was generally considered as a fatal one.

"It should be remarked that he ascribes the complaint to many other causes, besides the venereal disease, as to rheumatism, to gout, to cutaneous complaints; but notwithstanding this, he gave mercury, and mercury relieved them. Richerand* also, in his Nosographie Chirurgicale, considers the stricture of the rectum as sometimes arising from the venereal disease.

"If the relief of a complaint by mercury (added to the use of the tent or bougie), which is often accompanied by less equivocal symptoms of the venereal disease, and which has been considered as constantly fatal, be not thought sufficient to establish its syphilitic origin and nature, this opinion may, perhaps, derive some additional strength,

* Loco Citato.

† Le retrecissement de l'extremite inferieure du rectum est quelquefois un vice de conformation; mais plus souvent l'effet de l'epaississment venerienne de ses parois, de tous les symptoms de l'affection syphilitique il n'en est point de plus grave.—Nosographie Chirurgicale, Paris, 1808, tom. iii. p. 418.

when when we reflect how often the neighbouring parts are attacked with complaints, of which no practitioner doubts the venereal origin.

" The condylomatous excrescences, which we see every day surrounding the anus; the large rhagades in these parts, though not so frequent a disease, are known to be venereal. And these, also, in addition to the constitutional remedy, require a local treatment adapted to their nature, as well as the stricture of the rectum. Even the fistula in ano is sometimes the consequence of the venereal infection*, as has been remarked by Mr. Pott and Le Dran.

" After all, I do not by any means intend to assert, that it is always, or even most frequently, a symptom of the venereal disease. If it is met with in combination with other syphilitic complaints, there can be no doubt of the propriety of using mercury. And if it is relieved, in such cases, by mercury, added to the use of the bougie or tent, I think it is reasonable to employ, and to expect benefit from, the same means in other cases, where it may be the solitary symptom, and where we are disappointed in our other methods of cure."

*The Treatment of the Stricture of the Rectum*, is the subject of the third Section.

" It has frequently been remarked, says Mr. Copeland, that he who knows a disease cannot be much at a loss how to treat it; and although this may not be true, perhaps, in its fullest extent, I believe no one will dispute, that the art of curing diseases advances, in some sort of proportion to the increase of our pathological knowledge." From this commencement in the treatment, Mr. C. proceeds, grounding all his observations and practical science on the comparative treatment of former authors, and their elucidations of the disease. After having advanced thus progressively, in stating what has been done by others, and weighing in his own judgment what the result upon the whole should be, he closes the subject with the following aphorisms, and thus concludes the third Section.

" Before I quit this subject, it may not be unuseful shortly to recapitulate some of the most important circumstances of the disease and its treatment.

" First, That a stricture of the rectum is by no means so uncommon a disease as is usually imagined, and that it has been hitherto generally considered as necessarily fatal, because it has been discovered only in the last stages, or by dissection after death †.

" Secondly, That many of those obstinate cases of constipated bowels, which are of long duration, arise from an organic obstruction to the passage of the fæces, and that this obstruction is most frequently so situated as to be within the reach of surgical aid.

" Thirdly, That it is requisite, in such cases, to examine the anus with the finger, or if the symptoms be strongly marked, and there be no obstruction within the reach of the finger, to examine it with a rectum bougie.

---

* Pott's Works, by Earle, vol. iii. p. 87. Le Dran, Observ. de Chirurg, tom. ii. obs. 84.

† See some observations on the diseased appearances of the rectum, by Dr. Baillie.—Morbid Anatomy, page 111.

availing

"Fourthly, That the use of internal medicines alone will be unavailing in such cases, and that nothing without the use of the bougie affords any hope of relief to the patient.

"Fifthly, That the disease is much less frequently of a cancerous nature, than from the description of authors it may seem to be.

"Sixthly, That it is often combined with symptoms of the venereal disease, and, in such cases, is more readily relieved by mercury, added to the use of the bougie, than by any other means.

"Seventhly, That if the disease is often found in combination with syphilitic symptoms, it is fair to infer that it may, in some cases, also, be the solitary symptom, and that if it resist the local treatment, and there be reason to suspect venereal mischief in the habit, it is right to try the effect of the mercury at the same time.

"Lastly, That whatever be the nature of the disease, provided it be not true cancer, it is necessary to continue the use of the bougie, at intervals, for a considerable time after the free passage of the fæces has been established, and to return to it whenever there be any symptoms of a recurrence of the complaint."

"*The Fourth Section treats on the Hæmorrhoidal Excrescence.*"

Mr. Copeland very candidly states, that the subject of this section has been so very accurately described, and distinguished from other complaints, which it somewhat resembles, by Sir James Earle and some others; and is a disease so well known to surgeons, that it becomes unnecessary to enter into a minute detail of its nature and appearances. But he adds, that he who has no other opportunity of informing himself on this subject, than what is afforded him by the English writers, who have treated or touched on it, will be apt to conclude that the extirpation of such excrescences, either by ligature or excision, is always a safe and successful operation. Sir James Earle has powerfully urged, in his edition of the works of the immortal Percival Pott, the removal of them by ligature, and his cases were successful. And other surgeons have recommended excision, in cases of which the treatment has been also successful. But Mr. Copeland has found from practice that the operations by ligature and excision in some cases have proved fatal. He has not relied upon his own bare assertion for the verification of these facts; but has applied his knowledge and his reading to the establishment of his own opinion by that of others. *Petit* has related cases, where the plausibility of success *a priori* was the most promising, and where by ligature, the patients died. From such instances, in similar cases, if there be any system adopted for removing these obnoxious and vexatious excrescences without a chance of incurring such unexpected and dangerous consequences, certainly Mr. Copeland has done well, and given us a lively instance of the value of that activity of mind, which was thus awake to so laudable a duty. If ligature and excision be sometimes from an unforeseen state of the constitution of a patient liable to produce fatal consequences, surely that method which does not expose a patient to the same danger, is and must be acknowledged to be an improvement. And here an opportunity is given to remark that Mr. Copeland has displayed a great deal of that fire of genius which, when cultivated, must give us some idea of the character of what we expect from a true surgeon.

Mr.

Mr. Copeland has a just apprehension of the hazards that are incurred both by excision and ligature in these cases; and points out a middle course which promises to avoid the danger of both; in which he is sanctioned by the authority of Hippocrates.

"Among the various modes of extirpating the tumours, which are suggested or recommended by Hippocrates, who has described the disease and its cure almost as well as any of his successors, that of taking them off (I suppose pinching them off, ἀφελεῖν auferre,) with the fingers, deserves to be mentioned as a sort of medium between the ligature and the incision, comprehending, in cases where it is practicable, most of the advantages of each without the dangers of either. This rude operation of Hippocrates, (which he says may be done without telling the patient any thing of the matter,) or at least the essential principle of it, namely, of producing some kind of contusion at the time of removing the excrescence, is worthy of consideration. It is a well known fact that the instinct of animals directs them to bite off the umbilical chord, when they produce their young. The laceration which the parts suffer in this natural operation prevents all hæmorrhage from the chord. The life of the young is thus preserved, until the circulation is accommodated to that alteration which is necessary after birth, when the fœtus becomes a perfect and respiring animal."

Among other remedies to which Mr. Copeland has called our attention, is the frequent injection of cold water up the rectum, with the addition of a few grains of sulphate of zinc, as employed by Schmucker. He likewise relies much upon the application of bougies in this complaint, as practised by Monteggio, an eminent Italian surgeon.

Though the works of Hippocrates are open to every surgeon, yet the merit of bringing into notice a fact from this father of the art, has been left to Mr. Copeland; a fact that will probably supersede the employment of excision or ligature.

We think, and it is but barely a presumption, if our Author had pointed out that the common forceps, sharply dentated, would be the properest instrument to embrace the stem of the hæmorrhoid, so that by the lacerating action of it, any hæmorrhage would be prevented, that the direction would have been useful And as to ligature, none being made, no consequence from it could follow. The forceps should be applied to bring forward the hæmorrhoid, and on its division, all which is left within the gripe of the forceps will be sufficiently lacerated to stop all hæmorrhage.

The fifth Section treats of *Fistula in Ano.* The author very justly observes in a note, page 58:

"It is remarkable that a disease of so frequent occurrence should have excited so little of the attention of the English surgical writers. Fewer cases of the disease have been published, and fewer remarks on the infortunia and the anomalous circumstances attending it during the progress of the cure, than almost any other disease. Except the classical treatise of Mr. Pott, who has by no means exhausted the subject, there has hardly any thing appeared on this subject for the last century."

The point of this section goes chiefly to two circumstances, which render the operation of *Fistula in Ano* more complex and uncertain in its event. From the hæmorrhage which sometimes follows; and from the cause of the disease sometimes being too remote to be removed by the operation. The hæmorrhage has been known to be fatal. The stricture in the rectum with a morbid state of the inner membrane of the gut, is far from being less frequently fatal; and where this is the case, the fistula becomes only a symptom, or consequence of the original disease.

Our author says, " the degree of inconvenience therefore which the fistula produces, and the other circumstances of the more important disease, are to be taken into consideration before the operation be resolved on, in cases of this description.

And here also Mr. Copeland appeals to authorities which support his well intentioned improvement.

The latent sinuses which are to be found in the complex cases of fistula in ano, have not been amply treated on. A dependance on the excellent direction of Mr. Pott has given a confidence to the operative surgeon which makes him blind to any farther inquiry; but it must be remembered that the operation can do no more than relieve the cause within its scope; and that the operation is not the less valuable from not doing more than what Mr. Pott even intended it should do.

The remaining sections consist of well narrated Cases, illustrative of the Author's observations on the several diseases of which he treats. In conclusion, we may venture to recommend this work to the profession, as containing the most correct and clear account of a class of diseases often obscure, and too frequently neglected.

---

THE EDINBURGH MEDICAL AND SURGICAL JOURNAL, No. 24, FOR OCT. 1810.

Article I. " *An Essay towards an Inquiry how far the Effluvia from dead Animal Bodies, passing through the natural Process of Putrefaction, are efficient in the Production of malignant pestilential Fevers; and how far such Effluvia are capable "of exciting a putrefactive Emotion in all other' living ' animal Substances exposed to their action?'* " By C. CHISHOLM, M. D. F. R. S. &c. &c.

IN this very learned essay, Dr. Chisholm contends, " That the effluvia from dead animal bodies, passing through the natural process of putrefaction, and unrestrainedly diffused through the atmosphere, are injurious to living animal bodies exposed to their action, no more than in as much as their fœtor is offensive to the olfactory nerves; and that, when confined to a very limited space, and their principles, instead of entering into new combinations, are concentered, and in that state applied to, or received into, the bodies of living animals, these effluvia may act as a poison, producing, in the living animal frame, fever, perhaps, but incommunicable, or incapable of propagation by contagion;—or instant death, by a sudden exhaustion of the living principle." To support this opinion, the author does not trust to chemistry; he thinks, that to draw conclusions from the analysis which Chemists have given us of the gaseous

-ous products of putrefying animal substances; and to apply these conclusions to the investigation of the causes of diseases supposed to be their peculiar offspring, would be as unphilosophical as they would be fruitless. The clearest results would be only conjectural. He therefore deems it more rational to pursue his inquiry by stating facts: "facts obvious to the senses, evident to every understanding, presenting a clear and sensible line of discrimination, separating them from all the pretensions of chemical or theoretical speculation."

Dr. Chisholm then adduces a variety of interesting facts, from ancient and modern writers, as well as from his own experience. The conclusions which seem to result from the premises, which he forcibly and perspicuously states, he apprehends to be, 1. "That the theory of ingenious chemists, founded on experiments or speculations, to prove the pestilential influence of putrid animal effluvia, receives no support from practical knowledge, or the known economy of nature.

2. That in no known and well ascertained instance, are putrid animal exhalations productive of pestilential fevers.

3. That in a concentrated state, they either become a mephitic poison, producing asphyxia and instantaneous death, or seem to produce fevers of a malignant, indeed, but not pestilential nature.

4. That in every instance which seems hitherto to have been investigated, wherein putrid animal effluvia have been supposed to be the cause of epidemic malignant fever, other agents of a less dubious, better known, and well ascertained nature, exist, such particularly as marsh miasmata, and the exhalations from stagnant water, and damp unventilated places; and the types or forms of such epidemics thus attributed to putrid animal exhalations, are such as are known to be the peculiar product of marsh miasmata, being uniformly marked with exacerbations and remissions, or paroxysms and intermissions. Contagion and marsh miasmata, may act conjointly in the same person, but the character of each respective action is manifested; but when marsh miasmata and putrid animal effluvia are present at the same time, no action but that of the former is manifested—the latter exhibits none of a morbid character.

5. "That all, or almost all those manufactures, in which putrid animal exhalations are evolved, are no further injurious than by being nuisances by the fœtor which they emit.

6. "That as nuisances, but not as causes of disease, they should be as much as possible removed from the habitations of men.

7. "That putrid animal exhalations, which cannot be supposed to possess within themselves, or which have not, from the circumstances of the situation or manufacture in which they are evolved, a principle of decomposition, and a capacity of entering into new combinations; and are consequently the true uncombined product of animal putrefaction; are not injurious further than by their offensive fœtor.

8. "That very little probability exists that putrid flesh, rendered so by the natural process, which from selection, accident, or necessity, becomes the food, is injurious to the health of men; a provision being made in the gastric juice of the stomach, by which such food is rendered not only harmless but nutritious.

 9. "Viewing

9. "Viewing the whole of the subject, both in relation to the pestilential epidemics of brute animals, and of the human race, and inquiring into their reciprocal capacity of being communicable from the one class of animals to the other, I imagine there is sufficient evidence in our possession, that such capability does not exist; that the aura, or emanating gas from the diseased brute animal, does not produce disease of any description in a healthy human being exposed to it; that a human being feeding on, or receiving into his system, the flesh of a pestilentially diseased brute animal, may certainly have disease excited thereby in his system, but a disease of a nature altogether new, and terminating with him, either in cessation of disease or extinction of life; that the same series of cause and effect may take place, when brute animals feed on, or take into their system, the flesh of a pestilentially diseased human being: but that there is solid ground of belief, that such series of cause and effect is provided against by the instinct of the former, directing them to what is beneficial to, guarding them against what may be destructive of life.

10. "That as there is a necessity for the dissolution and reduction to their primary elements of animals, in order that the series of succession of animated nature may be maintained; so would it evince an unaccountable deviation from the compensative economy of God, should the process of that dissolution and reduction be productive of injurious effect, when the ultimate purpose of it is beneficial and compensative.

11. "That the dissolution and reduction to their primary elements of animals, may be considered as the physical link connecting the dead and the living, inanimate and animated nature—the one and the other mutually depending on each other, reciprocally contributing to each others support.

"*Finally*, that the result of the whole is, that in this, as in all things else, the wisdom, the mercy and goodness of God are manifested; that in this, as in all others, 'the works of the Deity are known by expedients;' for as the putrefaction of dead animal bodies is necessary to the accomplishment of the purposes of Divine Providence, for 'that which thou sowest is not quickened except it die;'—so the living, for whose existence it is, in a general sense, necessary, either do not experience it as an evil, are removed from, or have a compensation for it.'"

The second article presents us with an account of the endemical fever of Sicily, by Mr. Boyle, surgeon to the 62d regiment of foot. The symptoms varied little from those commonly described by authors; but the paper is highly valuable, from a successful mode of treatment being decidedly established. Two of the first patients died, and as the appearances on dissection enabled the author to form a more correct notion of the disease, and to determine upon the proper method of curing it, we shall insert his account of them. "On opening the scull, nothing uncommon was discovered on the external surface of the dura mater. Its sinuses, however, were gorged with blood, and the small veins, leading to the longitudinal one, much distended. Between the dura and pia mater, there was a small quantity of serum diffused. And the surface of both, in the neighbourhood of the longitudinal sinus, was covered with that sort of matter which is observed on the peritoneum and other membranous parts which have suffered from inflammation. Adhesions had had also taken place ; and in two or three places small abscesses were formed. In the ventricles, and within the substance of the brain, nothing uncommon was found. The dissection of the second case presented very similar appearances. The evidences of inflammation were indisputable ; in the first case, bleeding was not practised till the day preceding death, but even at that advanced period of the disease, instantaneous relief followed the insertion of the lancet. The second patient died without having lost any blood. Afterwards, when bleeding was resorted to in the first stage, not a single man, out of 240 who were attacked with fever, perished. This practice, in such cases, has been introduced into the army only recently, but the effects have proved highly beneficial.—Cold effusion and purgatives were also employed, but the remedy on which Mr. Boyle chiefly relied was copious bleeding, in the use of which he was directed by the headach and giddiness, rather than by the state of the pulse. He prefers taking the blood from the temporal artery. The Sicilian physicians generally applied leeches to the temples, and gave cold, acid, drinks. These facts are very important, and coincide with what we must regard as an improved mode of practice in this country, in the treatment of fevers. In the commencement, signs of inflammation are generally present, and by removing them, we often succeed in cutting short the fever, or at least in mitigating its violence and duration ; hence unquestionably is derived the good effect of cold effusion, of purgatives, and of local bleeding. In some instances, blisters applied to the head were of decided advantage, particularly when the giddiness which generally affected the patient, was protracted.

Art. 3, contains an Account of a *Disease of the Throat of an uncommon description, which occurred in the Hurrianah District.*

The first case is detailed by Lieutenant Higgins in a letter to Mr. Ramsay, Assistant Surgeon, 1st. Bat. 2d Regt. N. I. Bengal, dated Camp, Ballealee, 8th August, 1809, from which we extract the following particulars. "A sepoy, two days ago, apparently in high health, was found dead in his bed. No person being able to account for so sudden a death, it was attributed to the bite of a snake. Yesterday another man was seized in the following manner : He complained that he felt a severe pain in his throat, and died in the course of a couple of hours. Last night I was called up, another sepoy being taken ill. I immediately went to him ; he was able to give a distinct account of what he felt ; he complained of a dreadful pricking pain in his throat, which he felt gradually swelling ; and he began to feel great difficulty in breathing or speaking. He continued in this state till his voice went completely, when he lay down and died. He was not an hour and a half ill altogether, and did not survive ten minutes after his voice failed him ; his eyes were fixed from the first, which he complained of." There were no spasms about the neck, mouth, or temples, or any aversion to liquids. The three unfortunate sufferers were of different ages and casts : a Brahmin, a low cast Hindoo, and a Mussulman. It was not ascertained whether the two first had eat any vegetables during the day. The last man had not tasted a single thing the whole day previous to his death, it having been a day of some religious ceremony among the Brahmins, which forbid them them tasting food. The water at Ballealee is good, and the inhabitants are entirely ignorant of the disorder.

Mr. Ramsay states the case of a Khidmutgar boy who was attacked with a violent pricking pain in his throat, in a similar manner to the cases before related. "On the right side of the Os Hyoides, there was a considerable tension and hardness; his pulse was about 74, and of natural strength. He had no fever. In fact, in every other respect, he was perfectly well." A blister was applied, and he took ʒs. of the sulphat of zinc, which made him vomit immediately." After the vomiting, he said the prickles had gone out of his throat. Next morning he was perfectly well, and the tension and hardness in the throat were gone." Another case was cured with a dose of white vitriol. Mr. Ramsay had no opportunity of dissecting any case of this singular disorder; of the nature and cause of which he has been unable to form any idea. He thinks, however, that it resembles Cynanche Trachealis. Dr. Burt remarks, "that the symptoms bear a very great analogy to those of a disease prevalent, about twelve or eighteen months ago, among the horses of the Company's stud, by which many very fine animals were lost.— The Veterinary Surgeon found no remedy whatever. Horses we know cannot vomit. From the appearances on dissection, the Veterinary Surgeon pronounced the disease to be similar to the croup in the human species."

The 9th article describes with great accuracy, a *Case of Carditis, with the Appearances on Dissection.*

"A. B. On the 21st of April, was delivered of a fine healthy child. The labour was natural and easy; nothing uncommon occurred. On the following morning, I found her as well as I wished. In the course of the same day, however, whilst sitting up in bed, for the purpose of putting the child to the breast, she was suddenly seized with oppressive respiration, and complained of severe pain in the loins, particularly referable to the right side. When I saw her in the evening, I found her in a semi-recumbent posture; her breathing was difficult, short and oppressed; great anxiety pourtrayed in the countenance; pulse 130, small but regular; skin dry, but of a moderate temperature; tongue white and moist; severe pain in the loins. On the following day, the pain in the loins went off, and she now complained of severe oppression about the precordia; breathing short and oppressed, with severe pain at the back of the neck, and between the shoulders; occasional vomiting; pulse quick but regular. Blisters were applied, and a cathartic administered; blood was also taken away; but it exhibited no marks of inflammatory action, such as the buffy coat, or a firm, contracted, or cupped-like crassamentum. On the third day there was a considerable remission of symptoms, which lasted, however, only for a short time. They again returned with equal violence, and continued for two days, without any mitigation. On the sixth day, the pains entirely went away; her extremities became cold, and her whole body was bedewed with a cold, clammy perspiration;— pulse fluttering, and too quick to be counted; respiration frequent but comparatively easy, except that at intervals there was to be observed a convulsive catch, as it were, somewhat approaching to singultus; there was a superabundant secretion of mucus in the bronchiæ. In this state

I did

I did not expect her to remain many hours; but during the night she had some sleep, which she had not experienced since the attack; and awaking appeared relieved, her countenance having assumed more of the natural cast; pulse regular, about 100; breathing easy; complaining of no pain; stools frequent, and passed away involuntarily, as did her urine. She continued much in this state for three days, when her tongue became suddenly enlarged to thrice its natural size, so that it could not be kept within the teeth, and as hard to the touch as a board, and dry, which it had not been during the complaint. On the following day, which was the tenth from the attack, she died. On the following morning I examined the body.

On raising the sternum, there was observed, on the external part of the pericardium, a large patch of coagulable lymph, about four inches in circumference. On cutting into the pericardium the cavity was found to contain three ounces, or more, of a serous fluid; the heart was enveloped in a covering of coagulable lymph, about the eighth of an inch in thickness. The whole interior surface of the pericardium was also coated in the same way, and adhered strongly to the heart at its apex. This coat of lymph was easily separable, as it did not appear at all to adhere, except when the pericardium was attached to the heart. That portion of the left lung, which passes down by the pericardium, adhered to it through the medium of effused lymph; the lungs were sound, both on the right and left side; there were no adhesions with the pleura, in any parts of the chest, except at the anterior part of the pericardium, which was attached to the under surface of the sternum, and cartilages of the ribs over it. None of the abdominal viscera were in the least diseased; the uterus was about the size of a small melon, and perfectly healthy."

In his able report of diseases at the public dispensary, Dr. Bateman describes some cases of continued fever accompanied by a distressing and unmanageable affection of the bowels, while the head remained free from disorder. "Active purgatives, even when conjoined with opiates, appeared rather to augment than to mitigate the disorder of the bowels; nor did the neutral salts, or rhubarb, afford a more decided benefit; while on the other hand, opiates or astringents alone were manifestly injurious; giving rise to parched tongue, increased heat, and a more frequent pulse, without correcting the condition of the alvine excretions. In one or two cases, small doses of mercurials, as of the hydrargyrus cum creta, appeared to be beneficial, when repeated three times a day."

In two cases of tænia, the oil of turpentine was given in the large doses which have recently been most successfully prescribed in this capital. In the first patient, "it operated speedily as a purgative, without occasioning any sickness, or strangury, and expelled the worm: in the second, a worm of several feet in length had passed from the intestines, immediately before the patient applied to the dispensary, but considerable pain and uneasiness in the abdomen remained. She took an ounce and half of the oil of turpentine, which began to operate in the course of half an hour, and brought away four slimy stools, but without any worm: she has had no pain or uneasiness since that time. The remedy excited excited no pain or difficulty in passing the urine, and little thirst; but she was sick at each operation by stool, and felt considerable giddiness during the whole of the afternoon."

---

*Hortus Kewensis; or, a Catalogue of the Plants cultivated in the Royal Botanic Garden at Kew. By the late* William Aiton. *The second Edition, eelarged. By* William Townsend Aiton, *Gardener to his Majesty,* Vol. I. 8vo. *London,* 1810. *Longman, Rees, and Co. pp.* xl.—407.

With peculiar satisfaction we notice a new edition of the *Hortus Kewensis*. The relation that botany has to the art of medicine must render the re-publication of this work, become scarce, and of high price, extremely acceptable to those who know and appreciate the value of this auxiliary science. The original edition had the character of being useful and scientific beyond any thing of the kind, since the *Hortus Cliffortianus* of Linnæus. Mr. Aiton, in that edition, received the assistance of Dr. Solander while he lived, and afterward of Mr. Dryander. To these learned and acute botanists, the work is considered to be indebted for the whole of its science: all the specific characters, the collating of synonyms, the observations, the English names, the habitats, even the chronology, or the time of the introduction of various plants, are understood to have been furnished by these gentlemen. To this new edition the valuable assistance of Mr. Dryander is believed to be extended.

The *Hortus Kewensis* is professedly a compilation, but a compilation made under the eye of a master capable of supplying the desiderata, and knowing where to make an election. In its plan, the systematic arrangement laid down in Willdenow's edition of the *species plantarum*, is followed. All our modern periodical publications are regularly referred to, both for the sake of the English reader, for whose use the catalogue has been principally compiled, and to shew to those foreigners into whose hands it may fall, that Englishmen have not of late years been inattentive to the advancement of their favourite study.

We hope, in a future number, and when the remaining volumes come before us, (only the first volume is yet published), to go more distinctly into the merit of this valuable work; and which we have, from this specimen, no hesitation to recommend unreservedly to those of our readers, who either find delight in the science of Botany, or are at all sensible of the advantages that accrue to the theory and practice of medicine, from its study.

