# Errata

**Published:** 1810-11

**Authors:** 


					406, line 13 from bott. for >ve read he.

				

